# Reductive catalytic upcycling of polyethylene using 2-propanol as transfer hydrogen-donor source

**DOI:** 10.1016/j.isci.2026.116786

**Published:** 2026-07-16

**Authors:** Antonio Cosimo Pio Trimboli, Viviana Bressi, Emilia Paone, Riccardo Pellegrini, Paolo Lazzarini, Elena Groppo, Francesco Mauriello

**Affiliations:** 1 Dipartimento di Ingegneria Civile, Energia, Ambiente e Materiali (DICEAM), Università degli Studi Mediterranea di Reggio Calabria, Via Zehender (già via Graziella) - Loc. Feo di Vito, 89123 Reggio di Calabria, RC, Italy; 2 Chimet S.p.A., Via di Pescaiola n. 74, 52041 Viciomaggio, AR, Italy; 3 Department of Chemistry, NIS Centre and INSTM Università degli Studi di Torino, Via Quarello n. 15, 10135 Torino, Italy

**Keywords:** catalytic upcycling of polyolefins, polyethylene, hydrogen-donor alcohols, ru/Al_2_O_3_ catalyst, transfer hydrogenolysis/hydrocracking

## Abstract

The reductive upcycling of polyethylene (PE) promoted by a commercial Ru/Al_2_O_3_ catalyst under an inert atmosphere in the presence of hydrogen-donor alcohols is reported. Among the alcohols tested, 2-propanol (2-PrOH) enabled the highest PE conversion (65%) indicating that external molecular hydrogen is not required under the applied conditions. The process affords high selectivity toward C_12_–C_20_ liquid hydrocarbons with minimal gas formation. This previously unexplored approach applies transfer-hydrogenation concepts from biomass valorization to polyolefin upgrading, enabling efficient depolymerization without external molecular hydrogen.

## Highlights

• H_2_-free polyethylene upcycling using 2-propanol as solvent and *in situ* hydrogen donor
• Proof of concept: biomass transfer hydrogenation applied to polyolefin upgrading
• Commercial Ru/Al_2_O_3_ gives ∼65% PE conversion at 220°C in 1 h, mainly C12–C20 liquids
• Process validated using real post-consumer polyethylene waste feedstocks

## Graphical abstract

**Figure:**
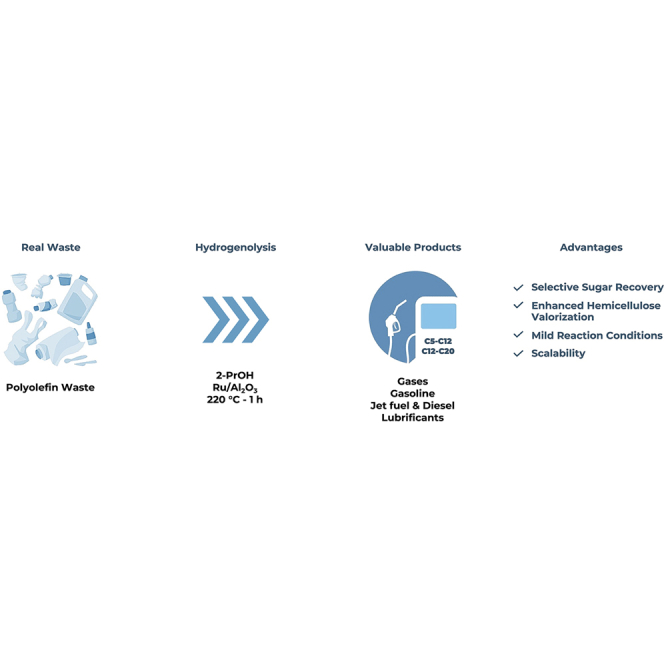

## Teaser

Environmental chemical engineering; Inorganic chemistry; Catalysis

## Introduction

Plastic waste is among the most pressing environmental and societal challenges of our time. By 2024, global plastic waste is projected to reach 6.3 billion metric tons with only 9% recycled, 12% incinerated, and the remaining 79% either landfilled or leaked into the environment.^1^^,^^2^ If current trends persist, this figure could exceed 12 billion tons by 2050.^3^ Major contributors to this crisis are polyolefins (POs)— including low-density polyethylene (PE), high-density PE, and polypropylene—which dominate the market due to their chemical inertness, low cost, large-scale production, and excellent mechanical and barrier properties. These materials account for nearly 50% of the global plastic demand with Europe alone consuming approximately 50 million tons annually. Despite their broad utility, over 95% of the POs are discarded after a short service life, contributing to more than 60% of the plastic fraction in municipal solid waste.^3^ Of this, only around 30% is currently recycled, while the remainder is incinerated, landfilled, or lost to the environment.^4^ To address this problem, chemical upcycling has emerged as a promising complement to mechanical recycling enabling the transformation of mixed or degraded plastic waste into fuels, monomers, or specialty chemicals.^5^^,^^6^^,^^7^ Among chemical approaches, thermochemical methods like pyrolysis are widely investigated but typically require high energy input and yield complex hydrocarbon mixtures with limited control over molecular weight distribution. Under such high-temperature conditions, heat and mass transfer are often limited by the viscosity and poor mobility of molten polymers.^8^ Olefin-metathesis strategies represent another distinct approach,^5^ circumventing direct hydrogenolysis by reorganizing carbon-carbon unsaturation through redistribution chemistry. Typically proceeding via a tandem sequence of dehydrogenation, olefin metathesis, and subsequent hydrogenation, these systems yield C_3_–C_20_ hydrocarbons under moderate temperatures (≈150°C–300°C) and near-ambient pressure. In contrast, reductive catalytic upcycling has recently gained attention as a more controlled alternative, enabling the transformation of POs into liquid alkanes with comparatively narrower molecular weight distributions under milder conditions.^8^^,^^9^ Catalysts based on Ru, Pt, and Ni supported on metal oxides have demonstrated significant activity for the reductive upcycling of PE.^10^ Catalytic performance depends on metal dispersion, support acidity, and metal-acid cooperativity. Notably, ruthenium (Ru) shows outstanding activity due to its low energy barrier for carbon-carbon bond cleavage.^11^^,^^12^ Among many, supports like alumina and zirconia, which offer moderate Lewis acidity and stabilize electron-deficient Ru species, often outperform carbonaceous supports in this context.^13^^,^^14^^,^^15^ Bifunctional systems, such as Ir/HBEA (Hydrogen Beta) zeolites, highlight the decisive role of Brønsted acidity in carbocation-mediated C–C bond scission under molecular hydrogen.^16^

Despite these advances, several challenges remain. Most reactions are conducted at high hydrogen pressures (20–60 bar) under solvent-free conditions (Table S1). This dependence increases process complexity, safety requirements, and capital costs. Even alternative “hydrogen-donor” strategies based on methanol or formic acid ultimately rely on the *in situ* generation of H_2_, and n-hexane and methanol have been explored as reaction media to improve polymer dissolution and reaction kinetics.^17^^,^^18^ Methanol, in particular, can serve as both reaction medium and hydrogen source via aqueous-phase reforming.^19^^,^^20^^,^^21^^,^^22^ Yet in these cases, the alcohol primarily functions as a precursor to molecular hydrogen, and the overall reaction mechanism remains indistinguishable from conventional hydrogenolysis.

Building on these insights and considering the well-established activity of Ru-based catalysts in the hydrogenolysis of POs under pressurized H_2_, we selected Ru as a rational catalytic platform for exploring alternative depolymerization strategies. Supported Ru nanoparticles have consistently shown superior activity for C–C bond cleavage in long-chain hydrocarbons and POs compared to many other transition metals, enabling the efficient conversion of these otherwise inert materials.^5^ In this context, we demonstrate a transfer-hydrogenolysis strategy for PE upcycling over a commercial Ru/Al_2_O_3_ catalyst operating under inert atmosphere, uniquely exploiting 2-propanol (2-PrOH) as both reaction medium and direct hydrogen donor. In contrast to previously reported alcohol-assisted systems, externally supplied molecular hydrogen is not required to sustain polymer depolymerization, and reaction performance shows no dependence on hydrogen pressure (*vide infra*). This strategy, inspired by biomass valorization where transfer-hydrogenation pathways are widely developed for selective depolymerization and upgrading,^23^^,^^24^^,^^25^^,^^26^^,^^27^ has never been systematically explored for the hydrogenolysis or hydrocracking of saturated POs. Importantly, while Ru-based catalysts are well known for PE hydrogenolysis under molecular hydrogen, their performance under transfer-hydrogenolysis conditions with alcohols is not established and cannot be assumed *a priori*. In conventional hydrogenolysis, surface hydrides originate from H_2_ dissociation, whereas under transfer-hydrogen conditions, hydrogen availability is governed by alcohol dehydrogenation kinetics and metal-acid interfacial processes. To establish proof of concept under industrially relevant conditions, a commercial 5 wt % Ru/Al_2_O_3_ catalyst was selected. This choice was not driven by catalyst novelty, but by its bifunctional properties and practical accessibility. Ru catalyzes both C–C hydrogenolysis and alcohol dehydrogenation, while alumina provides moderate Lewis acidity capable of stabilizing carbocationic intermediates. The resulting metal-acid synergy enables simultaneous alcohol-mediated hydrogen transfer and acid-assisted C–C bond activation, allowing operation in a transfer-hydrogenolysis regime distinct from conventional hydrogenolysis or strongly acid-driven hydrocracking.

## Results

### Characterization of the commercial Ru/Al_2_O_3_ catalyst

**Figure 1:**
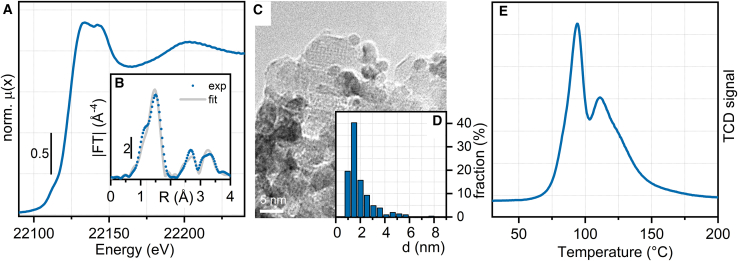
Physical-chemical characterization of the commercial Ru/Al_2_O_3_ catalyst employed in this work (A) Ru K-edge XANES. (B) Modulus of the k^3^-weighted EXAFS signal (dots) and best fit (gray line). (C and D) Representative HR-TEM picture (scale bar, 5 nm) and (D) corresponding particle size distribution. (E) H_2_-TPR profile.

The Ru/Al_2_O_3_ catalyst was prepared by deposition-precipitation^28^^,^^29^^,^^30^ and consists of highly dispersed RuO_2_-like nanophases supported on an industrial δ/θ-Al_2_O_3_ (surface area 121 m^2^ g^−1^; pore volume 0.43 cm^3^ g^−1^).^31^ Ru K-edge X-ray absorption spectroscopy (XAS) confirms a predominantly oxidic Ru environment (Figures 1A and 1B) consistent with a rutile-type RuO_2_ local structure, indicating that Ru is present as a supported RuO_2_-like nanophase rather than as metallic Ru passivated by a thin surface oxide.^32^^,^^33^ Additional extended X-ray absorption fine structure (EXAFS) fitting details, including phase-uncorrected Fourier transform spectra and individual Ru–O and Ru–Ru scattering contributions, are provided in Figure S1. High-resolution transmission electron microscopy (HR-TEM) (Figures 1C and 1D; Table S2) reveals a bimodal distribution of ultrasmall nanoparticles/clusters (∼1 nm) and minor crystalline domains (∼5 nm), while CO chemisorption after mild H_2_ pretreatment (120°C) indicates an Ru dispersion of ∼22%. Temperature-programmed reduction (H_2_-TPR) analysis (Figure 1E) demonstrates unusually facile reduction below 130°C via a defined two-step pathway (RuO_2_-like → Ru_2_O_3_-like intermediate → Ru^0^).^34^^,^^35^^,^^36^^,^^37^^,^^38^

These characteristics suggest that under reaction conditions, oxidic and partially reduced Ru motifs may coexist dynamically, particularly within the 100°C–130°C temperature regime identified by H_2_-TPR analysis. Such dynamic surface evolution may be especially relevant for transformations requiring controlled hydrogen availability and metal-acid interfacial cooperation. This picture is fully consistent with the two-step reduction pathway (RuO_2_-like → corundum-like Ru_2_O_3_ intermediate → metallic Ru^0^) recently elucidated by *in situ* synchrotron XAS, high-energy X-ray total scattering coupled with Pair Distribution Function (PDF) and chemometric PCA/MCR-ALS (Principal Component Analysis/Multivariate Curve Resolution - Alternating Least Squares) analysis for the same commercial Ru/Al_2_O_3_ catalyst employed in the present work.^31^ Further complementary physicochemical characterizations are currently under investigation to gain a more comprehensive understanding of the catalyst surface properties. In any case, rather than optimizing the catalyst composition at this stage, the primary objective of this study is to establish the chemical plausibility of alcohol-mediated transfer hydrogenolysis for PO conversion using an industrially accessible catalyst material. The successful conversion of both model and real PE substrates (*vide infra*) confirms the robustness of this system. Future studies will explore alternative catalysts (e.g., Ni, Pt, Pd, or stronger acid supports) to further optimize activity and selectivity.

### Effect of hydrogen donor and gas atmosphere on the reductive upcycling of polyethylene

At the beginning of our catalytic investigation, to evaluate the impact of the reaction medium on PE hydrogenolysis, H-donor screening was performed using PE4000 as a model substrate (PE).

**Figure 2:**
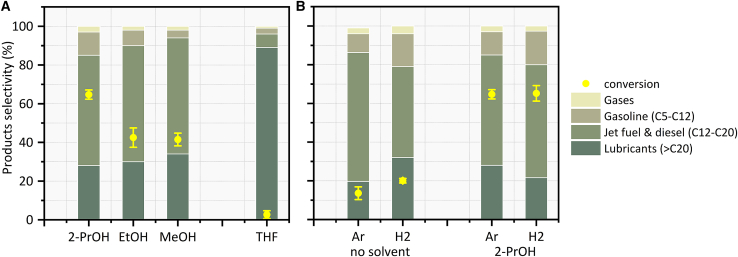
Role of hydrogen donor and gas atmosphere in the reductive upcycling of polyethylene (A) Effect of the H-donor source on the reductive upcycling of PE4000 promoted by the Ru/Al_2_O_3_ catalyst, in terms of both conversion and product selectivity (reaction conditions: PE4000 [0.222 g]; Ru/Al_2_O_3_ catalyst [0.111 g–5% of Ru], solvent [56 mL], temperature [220°C], reaction time [60 min], initial Ar pressure [30 bar], stirring rate [500 rpm], and vessel capacity [100 mL]). (B) Effect of the gaseous atmosphere (H_2_ vs. Ar), both in the presence and absence of 2-PrOH, on the reductive upcycling of PE4000 promoted by the Ru/Al_2_O_3_ catalyst, in terms of both conversion and product selectivity (reaction conditions: PE4000 [0.222 g]; Ru/Al_2_O_3_ catalyst [0.111 g–5% of Ru], solvent [56 mL], temperature [220°C], reaction time [60 min], initial Ar or H_2_ pressure [30 bar], stirring rate [500 rpm], vessel capacity [100 mL]). Polyethylene conversion values are reported as mean ± SD from three independent catalytic experiments (*n* = 3).

Reactions were conducted at 220°C for 60 min in a stainless-steel reactor under an initial argon pressure of 30 bar and a stirring rate of 500 rpm (Figure 2A; Table S3; Figures S2 and S3). The Ru/Al_2_O_3_ catalyst was used as received in a predominantly oxidic RuO_2_-like state, as indicated by XANES/EXAFS (X-ray Absorption Near Edge Structure/Extended X-ray Absorption Fine Structure) analyses. Under the adopted reaction conditions, partial *in situ* reduction of the oxidic Ru species is expected to occur, in agreement with the H_2_-TPR profile. Results reveal a striking H-donor dependence of both conversion efficiency and product distribution. Among the alcohols tested, 2-PrOH yielded the highest solid conversion (65%) and exhibited a well-balanced product distribution spanning gasoline-range (C_5_–C_12_) and jet fuel/diesel-range (C_12_–C_20_) hydrocarbons. This superior performance is consistent with the well-established behavior of secondary alcohols in transfer hydrogenation. Compared to methanol and ethanol, 2-PrOH undergoes more favorable dehydrogenation to acetone, facilitating hydride formation at Ru sites due to weaker α-C–H bonds. Gas chromatography (GC)-flame ionization detection analysis confirmed the formation of acetone as the primary product of 2-PrOH dehydrogenation over Ru sites, consistent with its role as hydrogen donor. Minor branched and etherification products were also detected (Figure S3), may be consistent with a bifunctional mechanism, in which PE is cleaved at Lewis-acidic alumina sites and hydrogenated via transfer from alcohol-derived hydrides. Importantly, no significant accumulation of gaseous hydrogen was detected under the adopted conditions. Semi-quantitative gas-phase analysis was performed in triplicate experiments and showed good reproducibility, with standard deviations below 4% across all tested conditions. Gas-phase products consistently represented only a minor fraction of the detected product distribution (approximately 1%–5%, depending on the reaction conditions), indicating limited gas formation under the adopted conditions. The description of the selectivity calculations was further clarified to specify that product distributions were obtained from normalized GC peak-area analysis considering detected products in both gas and liquid phases. These observations are fully consistent with our previous mechanocatalytic PE upcycling study performed under ambient temperature and atmospheric pressure conditions using the same Ru/Al_2_O_3_ catalytic system, where gas-phase formation likewise remained a minor component of the product mixture.^39^ While transient H_2_ formation arising from alcohol dehydrogenation cannot be entirely excluded, these observations indicate that hydrogen transfer occurs predominantly through surface-mediated pathways rather than through buildup of molecular hydrogen in the gas phase.

To further investigate the hydrogen source and hypothesized reaction mechanism, comparative experiments were conducted under H_2_ and Ar atmospheres, with and without 2-PrOH (Figure 2B; Table S4). In the absence of 2-PrOH (Figure 2B; Figures S4 and S5), where the polymer remains in a viscous melt, conversion was limited to 14% under Ar and only marginally improved to 20% under H_2_. In both cases, the product distribution skewed toward heavier hydrocarbons (C_12_–C_20_ and >C_20_), reflecting limited catalytic efficiency due to poor mass transfer and constrained hydrogenation dynamics. The introduction of 2-PrOH markedly enhanced the performance (Figure 2B). Under 30 bar H_2_, conversion reached 62% (Figure S6), whereas under 30 bar Ar, a slightly higher conversion of 65% was obtained, with nearly identical product distributions.

The absence of any performance enhancement under H_2_ pressure indicates that externally supplied H_2_ does not govern catalytic turnover under these conditions. Rather, hydrogen transfer from 2-PrOH appears sufficient to sustain C–C bond scission.

**Figure 3:**
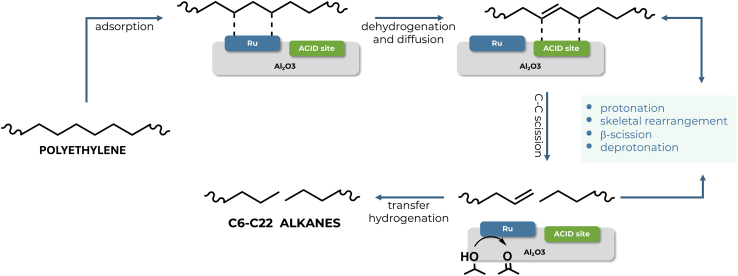
Simplified proposed reaction pathway in the reductive catalytic upcycling of PE4000 by using 2-propanol as transfer hydrogen-donor source

These results appear inconsistent with a mechanism dominated exclusively by classical hydrogenolysis,^40^ which typically involves dissociative H_2_ activation at metallic sites followed by direct surface hydride attack. Instead, the observed behavior may be consistent with a bifunctional regime in which C–C bond scission proceeds through carbocationic intermediates at Lewis-acidic Al_2_O_3_ sites, while Ru nanoparticles may mediate transfer hydrogenation using hydrogen species generated *in situ* from 2-PrOH dehydrogenation. Indeed, as schematically illustrated in Figure 3, Ru sites are expected to promote the initial adsorption and dehydrogenation of the alkane substrate, leading to the formation of olefinic intermediates. These unsaturated species can subsequently migrate toward the acidic sites of Al_2_O_3_, where protonation may generate carbocation-like intermediates. The latter can undergo skeletal rearrangement and β-scission, resulting in C–C bond cleavage and the formation of lighter olefinic fragments. At the same time, Ru sites are also proposed to play a key role in the final stabilization of these fragments through a hydrogen-transfer process. In particular, Ru-promoted transfer hydrogenation can convert the olefinic intermediates generated during cracking into the corresponding saturated hydrocarbons. Although further mechanistic studies are required to fully disentangle classical hydrogenolysis and hydrocracking contributions as well as to elucidate the individual elementary steps and the relative contribution of Ru and Al_2_O_3_, the lack of hydrogen-pressure dependence clearly distinguishes this system from conventional Ru-catalyzed hydrogenolysis.

### Reaction time and temperature optimization for the reductive upcycling of PE4000

**Figure 4:**
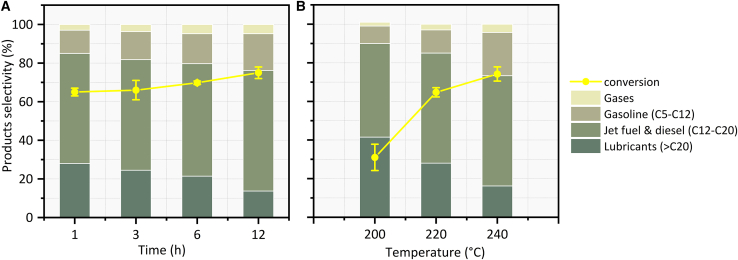
Optimization of reaction time and temperature for the reductive upcycling of PE4000 (A) Effect of reaction time on the reductive upcycling of PE4000 promoted by the Ru/Al_2_O_3_ catalyst, in terms of both conversion and product selectivity (reaction conditions: PE4000 [0.222 g]; Ru/Al_2_O_3_ catalyst [0.111 g–5% of Ru], solvent [56 mL], temperature [220°C], reaction time [1–12 h], initial Ar pressure [30 bar], stirring rate [500 rpm], vessel capacity [100 mL]). (B) Effect of temperature on the reductive upcycling of PE4000 promoted by the Ru/Al_2_O_3_ catalyst, in terms of both conversion and product selectivity (reaction conditions: PE4000 [0.222 g]; Ru/Al_2_O_3_ catalyst [0.111 g–5% of Ru], solvent [56 mL], temperature [200°C–240°C], reaction time [60 min], initial Ar pressure [30 bar], stirring rate [500 rpm], vessel capacity [100 mL]). Polyethylene conversion values are reported as mean ± SD from three independent catalytic experiments (*n* = 3).

Time-on-stream experiments at 220°C under 30 bar Ar with 2-PrOH demonstrated sustained catalytic activity over time under fully liquid-phase conditions, intentionally selected to minimize mass-transfer limitations and suppress gas-phase side reactions. Conversion increased from 65% after 1 h to 75% after 12 h (Figure 4A), accompanied by a progressive shift from >C_20_ lubricants toward lighter jet and gasoline fractions (Table S5; Figure S7), indicating ongoing C–C bond cleavage and secondary cracking. Importantly, gas formation remained limited throughout the investigated reaction times, <5% of detected products, indicating sustained selectivity toward liquid hydrocarbons. Complete conversion was not achieved even after prolonged reaction times. This behavior is consistent with literature reports on PE hydrogenolysis under batch conditions,^41^^,^^42^^,^^43^ where waxy intermediates and viscous residues can partially encapsulate catalyst particles and limit polymer accessibility. These physical constraints, rather than catalyst deactivation, are therefore likely responsible for incomplete conversion. Importantly, gas formation remained consistently low throughout, underscoring the high selectivity toward liquid hydrocarbons.

We next assessed the effect of temperature under identical conditions (Figure 4B; Table S6; Figures S8 and S9). At 200°C, conversion was modest (31%) and the product slate was dominated by heavy hydrocarbons (42% > C_20_), with jet fuel range compounds accounting for 48% and gasoline-range products limited to just 9%. Limited gas formation (∼1% of detected products) suggests that chain scission events were infrequent at this temperature under the adopted reaction conditions. As previously discussed, raising the temperature to 220°C significantly improved the conversion to 65% and shifted the product distribution toward lighter hydrocarbons: the lubricant fraction dropped to 28%, jet fuel range peaked at 57%, and gasoline range rose to 12%. This improvement reflects enhanced polymer mobility and more efficient C–C cleavage. At 240°C, the conversion reached 74%, accompanied by a further decline in the lubricant fraction (17%) and a rise in gasoline-range products (22%). The jet fuel range remained stable at 57%. Gas formation increased to 4%, and the appearance of lighter products indicates the onset of overcracking, likely due to excessive thermal input. In any case, the relatively low formation of gaseous products, <5% under all investigated conditions, rather supports the hypothesis that the reaction follows a hydrocracking mechanism. Negligible wax formation was observed across the entire temperature range investigated. Overall, these results collectively indicate that 220°C represents a good trade-off between conversion efficiency and product selectivity, maximizing the yield of desirable liquid hydrocarbons while minimizing unwanted gas formation and overcracking.

### Extension to real plastic waste feedstocks

**Figure 5:**
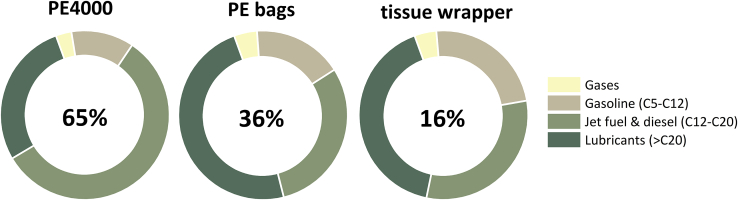
Performance of Ru/Al_2_O_3_ in the reductive upcycling of model and real polyethylene feedstocks Comparison of reductive catalytic upcycling of model PE4000 and real plastic waste (fresh vegetable polyethylene bags and tissue wrappers) promoted by Ru/Al_2_O_3_ catalysts, under Ar atmospheres, in the presence of 2-PrOH as H-donor source (reaction conditions: POs [0.222 g]; Ru/Al_2_O_3_ catalyst [0.111 g–5% of Ru], solvent [56 mL], temperature [220°C], reaction time [60 min], initial argon pressure [30 bar], stirring rate [500 rpm], vessel capacity [100 mL]). Polyethylene conversion values are reported as mean ± SD from three independent catalytic experiments (*n* = 3).

To explore the applicability of the method beyond model polymers, two different post-consumer plastic waste samples were evaluated (Table S7; Figure S10) under the optimized conditions (Ru/Al_2_O_3_, 220°C, 1 h, 2-PrOH, Ar). The results, summarized in Figure 5, demonstrate that the system retains catalytic activity even in the presence of complex, unrefined substrates, highlighting its intrinsic robustness and potential for practical applications. All experiments were conducted on a gram scale (typically 0.5–1.0 g of polymer per run), including trials with post-consumer plastics. Despite the complexity of these substrates, due to additives and multilayer structures, the catalyst remained active and selective, confirming the adaptability of the system beyond idealized polymers. When PE-based plastic bags were used, a solid conversion of 36% was achieved. Although primarily composed of PE, the presence of additives and/or copolymers can influence solubility and catalyst interaction. Nevertheless, the product distribution is dominated by lubricants (>C_20_, 48%) and gasoline range fractions (31%) with minimal gas production (4%). Tissue wrappers, which typically consist of multilayer films, coatings, and pigments, yielded a lower conversion (16%), as expected. However, the formation of gasoline-range products (31%) and partial depolymerization into lubricant-range hydrocarbons (24%) indicate that catalytic activity is retained and that further optimization could unlock higher efficiencies. Rather than highlighting the limitations, these findings underscore the versatility and adaptability of the system. The observed catalytic activity toward heterogeneous plastic waste, even in the presence of additives and structural complexity, highlights the conceptual versatility of the system and motivates further investigation toward more robust and scalable chemical upcycling strategies. These insights pave the way for future developments, including tailored catalyst formulations and targeted pre-treatment strategies, to further enhance performance across diverse waste streams.

## Discussion

This work demonstrates the feasibility of alcohol-mediated transfer hydrogenolysis for PE upcycling under inert atmosphere using a commercial Ru/Al_2_O_3_ catalyst and 2-PrOH as both reaction medium and hydrogen donor. In contrast to conventional hydrogenolysis systems, catalytic turnover proceeds without reliance on externally supplied molecular hydrogen and no enhancement is observed under hydrogen pressure. These findings support operation in a transfer-hydrogen regime conceptually distinct from the classical H_2_-driven hydrogenolysis.

The system affords high selectivity toward liquid hydrocarbons with minimal gas formation, and product distribution can be tuned through temperature and time. The ability to maintain catalytic activity in the presence of post-consumer PE-based waste further demonstrates operational robustness under realistic substrate complexity.

Mechanistically, the results appear consistent with a bifunctional metal-acid pathway in which alcohol dehydrogenation at Ru sites supplies surface hydrogen equivalents, while Lewis-acidic alumina facilitates carbocation-mediated C–C bond cleavage. Although additional studies are required to fully resolve the relative contributions of hydrogenolysis and hydrocracking pathways, the absence of hydrogen-pressure dependence clearly differentiates this system from conventional Ru-catalyzed hydrogenolysis.

This work introduces a fundamentally new paradigm for PO upcycling by demonstrating, for the first time, that simple aliphatic alcohols can act as direct hydrogen donors for PE depolymerization, enabling efficient conversion in the absence of externally supplied H_2_. This hydrogen-free strategy simplifies process requirements, improves operational safety, and allows the reaction to proceed under comparatively mild conditions with high selectivity toward liquid hydrocarbons and negligible gas formation.

A central conceptual advance is the direct translation of transfer-hydrogenation approaches from biomass valorization to PO conversion. This connection substantially broadens the catalytic toolbox available for plastic upcycling, indicating that catalytic systems and mechanistic concepts developed for lignocellulosic substrates can be effectively adapted to POs. As such, this study establishes a framework that can facilitate rapid exploration of new catalyst formulations and reaction pathways, potentially accelerating progress across both research areas.

Within this context, several aspects merit further investigation. The mechanistic picture, although consistent with a transfer-hydrogenolysis regime, requires deeper clarification. In particular, the nature of hydrogen transfer, the role of metal-acid cooperativity, and the relative contributions of hydrogenolysis and hydrocracking pathways remain to be elucidated through dedicated kinetic studies and operando characterization. From a catalytic standpoint, the system shows good activity and selectivity, but long-term stability and recyclability have not yet been systematically assessed. Ongoing work is directed toward the development of thermal regeneration strategies aimed at restoring the initial physicochemical properties of spent catalysts, thereby enabling their reuse over multiple cycles without loss of performance.

The applicability to real waste streams represents an important outcome of this study, as catalytic activity is maintained even in the presence of post-consumer PE. At the same time, the influence of impurities, additives, and multilayer structures on catalytic behavior and product distribution requires further evaluation. A systematic analysis of these effects will be necessary to define the robustness of the process across heterogeneous and contaminated feedstocks.

Process development aspects also remain to be addressed. Although the current results demonstrate feasibility at the laboratory scale, further work will focus on scale-up, including reactor design, transport phenomena, and operation under continuous or semi-continuous conditions. These studies will be essential to assess process intensification and practical implementation.

Overall, the present study provides a proof-of-concept conceptual framework for exploring hydrogen-free PO upcycling strategies based on alcohol-mediated hydrogen transfer. The combination of operational simplicity, compatibility with real waste, and conceptual linkage to established biomass conversion strategies highlights significant potential for further development and optimization.

### Limitations of the study

The present study provides a proof-of-concept demonstration of alcohol-mediated catalytic transfer hydrogenolysis/hydrocracking for PE upcycling under inert atmosphere. Although catalytic activity was demonstrated for both model PE and post-consumer plastics, conversion yields from real waste remain moderate and the system has not yet been systematically optimized. Catalyst stability, recyclability, and long-term performance were not investigated, while the effects of additives, multilayer architectures, and contaminants require further study before scalability and practical applicability can be reliably assessed. Further complementary physicochemical characterizations are also underway to provide a more comprehensive understanding of the catalyst surface properties and the nature of the active sites involved in the reaction.

The proposed mechanism, involving alcohol-mediated hydrogen transfer combined with metal-acid cooperativity and carbocation-mediated C–C bond scission, is consistent with the experimental evidence but should be regarded as a working hypothesis rather than a definitive assignment. This study prioritized proof-of-concept feasibility over mechanistic resolution; consequently, isotopic labeling, rigorous hydrogen/carbon balance analyses, operando spectroscopy, and systematic hydrogen-donor control experiments were deferred. The respective contributions of transfer hydrogenolysis, hydrocracking, and transient molecular hydrogen, therefore, remain to be clarified and will be addressed in future work.

The reported product distributions are also subject to the analytical limitations associated with complex hydrocarbon mixtures from polymer depolymerization. Liquid-phase selectivities are based on normalized GC peak areas and are intended mainly for comparative evaluation rather than as absolute molar yields. Gas formation remained below 5% and wax formation was negligible under all conditions, although high-boiling fractions not fully accessible to GC analysis make the distributions semi-quantitative. Likewise, unequivocal attribution of hydrocarbons to PE- or 2-PrOH-derived pathways is not possible without isotopic tracing. Control experiments indicate that PE is the predominant carbon source, but rigorous carbon-balance closure and product-origin mapping will require dedicated future studies.

## Resource availability

### Lead contact

Requests for further information and resources should be directed to and will be fulfilled by the lead contact, Prof. Francesco Mauriello (francesco.mauriello@unirc.it).

### Materials availability

All reagents in this study are either commercially available or can be prepared as indicated in the supplemental information.

### Data and code availability

• All data generated or analyzed during this study are included in this published article and its supplemental information files.
• This study did not generate any custom code. Any software or analysis tools used are standard and are cited appropriately in the STAR Methods section. Therefore, no code was deposited in an external repository.
• This study did not generate new unique reagents, materials, or other resources. All materials used are described in detail in the STAR Methods section.

## Acknowledgments

This publication was prepared with support and funding from the Italian Ministry of University and Research (MUR) within PRIN 2022 project “2022PPXWMS - Catalytic Upcycling of PolyolefIn waste: a matter of awareness (CUPId).” P.L. and E.G. acknowledge support from Project CH4.0 under the MUR program Dipartimenti di Eccellenza 2023 2027 (CUP D13C22003520001).

## Author contributions

E.G. and F.M conceived and supervised the project; R.P. prepared the Ru/Al_2_O_3_ catalyst; E.G. and P.L. carried out the physico-chemical characterization; A.C.P.T. and V.B. performed the catalytic tests; A.C.P.T., E.P., E.G., and F.M. co-wrote the manuscript original draft, ; A.C.P.T. and F.M. were responsible for manuscript revision and editing. All authors have read and agreed to the published version of the manuscript.

## Declaration of interests

The authors declare no competing interests.

## Declaration of generative AI and AI-assisted technologies in the writing process

During the preparation of this work, the authors used ChatGPT (GPT-5.5, OpenAI) in order to improve the writing style. After using this tool/service, the authors reviewed and edited the content as needed and take full responsibility for the content of the publication.

## STAR★Methods

### Key resources table

**Table:** 

REAGENT or RESOURCE	SOURCE	IDENTIFIER
**Chemicals, peptides, and recombinant proteins**		

Ru/Al_2_O_3_	Chimet	7440-18-8
Polyethylene	Sigma-Aldrich	9002-88-4
PE bags	Real waste	N/A
PE Tissue wrapper	Real waste	N/A
MeOH	Sigma-Aldrich	67-56-1
EtOH	Sigma-Aldrich	64-17-5
2-PrOH	Sigma-Aldrich	67-63-0
THF	Sigma-Aldrich	109-99-9
Argon	Air Liquide Italia	7440-37-1
Hydrogen	Air Liquide Italia	1333-74-0

**Software and algorithms**		

Demeter(Athena and Artemis software package)	N/A	N/A

**Other**		

Autoclave Reactor	Parr Instrument Company	Parr 4848
GC-MS	Shimadzu	GCMS-QP2010
GC	Shimadzu	GC-2014
Microscope	Jeol USA	3010-UHR microscope
Temperature-programmed chemisorption	Micromeritics	Autochem 2910

### Method details

#### Catalyst synthesis

The 5wt % Ru/Al_2_O_3_ catalyst employed in this study was synthesized at the Catalyst Division of Chimet S.p.A. using a deposition-precipitation approach. RuCl_3_ served as the metal precursor, while Na_2_CO_3_ was employed as the precipitating agent. A commercial alumina was used as support (surface area: 121 m^2^/g; pore volume: 0.43 cm^3^/g; composed of δ and θ phases). After the deposition-precipitation, the catalyst was reduced in NaBH4, and then exposed to air, filtered, thoroughly washed with water to remove residual chloride ions and subsequently dried at 90°C overnight.

#### Characterization of the Ru/Al_2_O_3_ catalyst

High-resolution transmission electron microscopy (HR-TEM) was performed using a JEOL 3010-UHR microscope, featuring a LaB_6_ electron source operating at 300 kV and a Gatan US1000 CCD camera (2 k × 2 k pixels). The Ru/Al_2_O_3_ sample was deposited onto a copper grid covered with lacey carbon film, avoiding the use of solvents to prevent potential reactions with the Ru-phase. To evaluate the particle size distribution, a total of more than 100 particles were manually counted. Bin widths for the histogram were determined using the Rice rule estimator to ensure statistically meaningful grouping.^44^ The geometric dispersion was calculated directly from the TEM-derived size distribution. assuming spherical geometry for the nanoparticles.

The metal dispersion was estimated via geometrical analysis of the HR-TEM particle size distribution, assuming spherical Ru nanoparticles. For each diameter class, the number of surface and total atoms was calculated using established power-law relationships based on the atomic radius of Ru (0.265 nm). The overall dispersion was then computed as the ratio of the weighted sum of surface atoms to total atoms across all size classes. This method accounts for the full distribution rather than relying on average size, ensuring a more accurate and reproducible estimation. In particular, the method assumes that Ru nanoparticles are approximately spherical, and uses established relationships between particle diameter (*d*), the total number of atoms (*N*_*TOT*_) and the number of surface atoms (*N*_*surf*_). In particular, the particle diameter *d* can be expressed as a function of the *N*_*TOT*_ and *N*_*surf*_ according to power laws of the form:

$$d=a{N_{TOT}}^{b}$$

$$d=c{N_{surf}}^{d}$$

where a, b, c and d are constants determined by the atomic diameter (for Ru approximated at 0.265 nm).

The particle size distribution from TEM was expressed as a histogram with percentage fractions *x*_*i*_ for each diameter class *d*_*i*_. For each class *d*_*i*_ the corresponding *N*_*TOT*,*i*_ and *N*_*surf*,*i*_ were calculated by inverting the above power laws. The overall metal dispersion *D*(%) was then computed as:

$$D\left(\%\right)=\frac{\sum_{i}x_{i}N_{surf,i}}{\sum_{i}x_{i}N_{TOT,i}}$$

Unlike methods that rely on a single average particle size, this procedure accounts for the entire particle size distribution, ensuring that both small and large particles contribute proportionally to the final dispersion value.

Temperature-programmed reduction (H_2_-TPR) experiments were conducted using a Micromeritics Autochem 2910 instrument equipped with thermal conductivity detectors (TCD) and a liquid nitrogen cryocooler for sub-ambient temperature control. A molecular sieve trap was placed between the sample bed and the detector to capture water generated during reduction. Calibration of the detectors was performed using a CuO standard to quantify hydrogen uptake. Approximately 100 mg of catalyst were loaded into a quartz reactor and purged with argon (50 mL/min) at room temperature for 5 min to remove weakly adsorbed species. The temperature was then lowered to −70°C, and the sample was exposed to a 5% H_2_/Ar mixture (50 mL/min) for 35 min to stabilize the signal and temperature. Subsequently, the temperature was ramped to 400°C at a rate of 5 °C/min. Quantification was based on the assumption that RuO_2_ is the predominant initial phase, with sample weight corrected for water content as determined by thermogravimetric analysis (TGA) up to 1100°C.

X-ray absorption spectroscopy (XAS) at the Ru K-edge was performed in transmission mode at the ID24 DCM beamline of the European Synchrotron Radiation Facility (ESRF) in Grenoble. The incident white beam was monochromatized using a fixed-exit double-crystal monochromator equipped with Si(111) and Si(311) crystals. Harmonic rejection was achieved via two mirrors positioned between the monochromator and the undulator.^44^ Detection was carried out using three ionization chambers: the first measured the incident beam intensity, the second recorded the transmitted signal through the sample, and the third monitored a metallic Ru reference foil to ensure accurate alignment of the absorption edge. Spectra were collected over the energy range of 21800–23200 eV, with a step size of 0.5 eV and a total acquisition time of approximately 7 s per spectrum. Data processing, including energy calibration and normalization, was conducted using the Athena software package.^45^^,^^46^ Extended X-ray absorption fine structure (EXAFS) analysis was performed with Artemis.^46^^,^^47^ The k^3^-weighted χ(k) functions were Fourier transformed within the k-range of 3–15 Å^−1^, and fitting procedures were carried out in R-space over the interval 1 to 4 Å.

#### Catalytic tests

The catalytic upcycling of POs was carried out through a discontinuous batch system. All reactions were conducted in triplicate to ensure reliability and accuracy of results. Unless otherwise specified, all experiments were carried out under identical conditions (Ru/Al_2_O_3_ catalyst, 2-propanol as hydrogen donor/solvent, Ar atmosphere at 30 bar, 220°C, 500 rpm). In a typical experiment, commercial polyethylene (PE4000) was mixed with the Ru/Al_2_O_3_ catalyst at a mass ratio of 2:1, along with 56 mL of the selected solvent. The mixture was loaded into a 100 mL stainless steel autoclave (series 4848 Parr Instrument Company, IL, USA). The reactor was heated at a rate of 3 °C/min until reaching the target temperature, at which point the reaction time was initiated. The process was conducted under constant stirring (500 rpm). Prior to pressurization, the reactor was purged five times with argon to remove residual air and then pressurized to the desired value with argon. Upon completion of the reaction, the system was cooled to room temperature in an ice bath. The liquid phase was separated from the solid residue by vacuum filtration.

#### Product determination and quantification

After the reaction, the liquid phase was treated with an antisolvent and left to stand overnight to allow precipitation. The resulting sediment was attributed to non-detectable waxes, while the filtrate was considered to contain unconverted polymer. The amount of unreacted substrate was estimated by subtracting the catalyst mass from the total mass of the recovered solid. This solid was then dried in an oven at 80°C overnight. The conversion of PO was determined using the following formula:

$$Conversion\;of\;POs=1-\left[\frac{m_{\left(unreacted\;POs\right)}-m_{\left(catalyst\right)}}{m_{\left(initial\;POs\;mass\right)}}\right]\;x\;100$$

where *m*_*(unreacted POs)*_ is the mass of the dried residue after solvent removal, and *m*_*(initial POs mass)*_ is the initial mass of plastic introduced into the reactor.

In accordance with the literature, only products with carbon numbers up to C45 – detectable by GC-MS – were considered for analysis, due to the high boiling points of heavier compounds. These were categorized into three fractions: C5–C12 (gasoline range), C12–C20 (jet fuel range), and >C20 (lubricant range).^48^ Waxes were quantified by adding a counter-solvent (acetone) to the filtrate, promoting the precipitation of any dissolved high-molecular-weight species. The resulting solid was collected by vacuum filtration and dried overnight at 80°C before weighing. Liquid products were analyzed by GC-MS using a GCMS-QP2010 Ultra system (Shimadzu, Japan) equipped with a split–splitless injector. For each GC analysis, a weakly polar HP-5 capillary column (30 m × 0.25 mm i.d. × 0.32 μm) was used. Helium was employed as the carrier gas, maintained at a constant flow rate of 25 mL/min. For system configuration, 5 μL of the sample was injected in split mode. The oven temperature was initially set at 50°C (held for 1 min), then increased up to 250°C (held for 10 min) at a heating rate of 20 °C/min. The mass spectrometer operated in electron ionization mode at 70 eV, and mass spectra were collected over a molecular mass range (m/z) from 40 to 400. Compound identification was performed by comparing the spectra with those from the US National Institute of Standards and Technology (NIST, ver. (11) mass spectral library. Overall product accounting included recovered solid residue, precipitated wax fractions, GC-detectable liquid products, and gaseous products. A rigorous absolute carbon balance was not performed. Due to the intrinsic complexity of post-reaction mixtures and the presence of high-boiling heavy fractions not fully detectable by GC analysis, the reported product distributions should be interpreted as semi-quantitative product accounting suitable for comparative evaluation of catalytic performance rather than as absolute carbon closure.

#### Product determination and quantification in solid-solid state reaction

For the solid-solid analysis, the reaction was performed at 220°C for 60 min at pressures of 30 bar of argon or hydrogen. At the end of the reaction, the reaction product formed a viscous residue, which was subsequently solubilized in isopropanol for 20 min at 50°C. The recovered product was analyzed following the same protocol as applied to the other reactions.

#### Gaseous phase determination and quantification

According to Chew et al., after the reaction, the reactor was connected to a gas chromatograph Shimadzu GC-2014 equipped with both a thermal conductivity detector (TCD) and a flame ionization detector (FID) for analysis of the gas-phase product samples. Hydrogen and CH_4_ were quantified using the TCD, while light hydrocarbon gases C_2+_ were analyzed using the FID.^49^ The columns included a right 12.5 m (L) × 0.32 mm (i.d.) packed column, which comprised 3 m Hayesep D, 4 m HS, and 2.5 m HN, and a left 2 m (L) × 0.32 mm (i.d.) 10% Carbowax 20 M Ch packed column. For these light hydrocarbons, carbon-number regions were assigned based on the expected chromatographic retention behavior under the applied analytical conditions, and product distributions were interpreted according to the anticipated elution trend of homologous *n*-alkane series.

#### Selectivity calculation

The selectivity of each product was determined based on GC peak areas, considering both liquid- and gas-phase products. Liquid products were analyzed by GC-MS, while gaseous products were analyzed by GC-FID. For light hydrocarbons, carbon-number regions were assigned according to the expected chromatographic retention behavior under the applied analytical conditions. Due to the complexity of the hydrocarbon mixtures and the absence of compound-specific calibration standards for all detected species, GC-derived product distributions should be regarded as semi-quantitative relative distributions rather than absolute molar yields. Accordingly, the reported selectivities are intended primarily to compare relative trends among experimental conditions. Liquid- and gas-phase product distributions were determined through normalized GC peak-area analysis. The selectivity for a given product i was calculated using the following equation:

$$Selectivity=\frac{A_{i}}{\Sigma A_{j}}\times 100$$

where A_i_ is the GC peak area of product i, and ∑A_j_ is the total area of all identified and quantified products in both gas and liquid phases.

### Quantification and statistical analysis

All catalytic experiments were performed in triplicate (*n* = 3 independent reaction experiments), unless otherwise stated. Polyethylene conversion values are reported as mean ± standard deviation (SD), where the mean represents the measure of central tendency and the SD the measure of dispersion. Mean values and standard deviations were calculated using Microsoft Excel. No inferential statistical tests were applied, as the study was designed to provide descriptive comparisons of catalytic performance rather than to test hypotheses. Selectivity values were obtained from the analysis of normalized GC peak areas, considering all products detected in the liquid and gas phases. Given the complexity of the hydrocarbon mixtures and the absence of specific calibration standards for each detected species, these values are reported as semi-quantitative relative distributions and are not expressed as mean ± SD or as absolute molar yields. Statistical details—including the exact value of n, the definition of n, measures of central tendency and dispersion, and data presentation methods—are provided in the “method details” section, the Supplementary Information, figure captions, and, where appropriate, the “results” section.
