# Supplementary material for: Reductive catalytic upcycling of polyethylene using 2-propanol as transfer hydrogen-donor source
Source: iScience. 2026 Jul 16;29(8):116786. doi: 10.1016/j.isci.2026.116786 (PMC13400963; doi:10.1016/j.isci.2026.116786)
Supplement: Document S1. Figures S1–S10 and Tables S1–S7 [file mmc1.pdf]

iScience, Volume *29*

Supplemental information

Reductive catalytic upcycling of polyethylene using 2-propanol as transfer hydrogen-donor source

Antonio Cosimo Pio Trimboli, Viviana Bressi, Emilia Paone, Riccardo Pellegrini, Paolo Lazzarini, Elena Groppo, and Francesco Mauriello

SUPPLEMENTAL INFORMATION

Table of Contents

1. Literature survey
2. Experimental Section
3. Supplemental References

1. **Literature survey**

Table S1. Overview of some of the reported catalytic upcycling reactions of polyolefins (POs) using Ru/Al_2O_3-based catalysts. Key reaction parameters (time, temperature, H_2 presure, polymer:catalyst molar ratio), conversion, product distributions and yields are summarized.

Catalyst	Polyolefin	Reaction Conditions				Conversion	Products Yields (%)			Ref.
		Time (h)	Temperature (°C)	H_2 pressure (bar)	P:C		gas	liquid	wax	
**5%Ru/γ-Al_2O_3**	LDPE	4	240	60	34:1	86%	2.6	62	21	Tamura et al. 2022[1]
**5%Ru/γ-Al_2O_3**	LDPE	4	240	60	34:1	>99%	26	70	4.8	Tamura et al. 2022[1]
**5%Ru/γ-Al_2O_3**	*n*-octadecane	14	200	50	26:1	65%	ND	ND	ND	Rorrer et al. 2021[2]
**5%Ru/γ-Al_2O_3**	*n*-octadecane	14	250	50	26:1	100%	ND	ND	ND	Rorrer et al. 2021[2]
**5.7%Ru/γ-Al_2O_3**	PP	4	240	20	5:1	70%	20	15.4	ND	Jaydev et al. 2023[3]
**5%Ru/γ-Al_2O_3**	LDPE	3	250	30*	34:1	44.2	8	32.7	3.5	Kwon et al. 2024[4]
**1%Ru/Al_2O_3**	PE	4	240	40	13:1	60.8%	ND	96.4	ND	Mei et al. 2024[5]

*Coreactant: water, water/C: 1:1

2. Experimental Section

2.1. $RuAl_2O_3$ catalyst characterization

Figure S1 shows the Ru K-edge EXAFS spectrum of pristine Ru/Al_2O_3, along with the best fit curve and the four scattering paths adopted for the fitting procedure. The first coordination shell (Ru-O) was successfully modelled with approximately six oxygen neighbours, distributed over two closely spaced distances, consistent with the structural features of RuO_2. Additionally, two distinct peaks are observed in the |FT| of the EXAFS signal (around 2.6 and 3.2 Å, not phase-corrected). These features are attributed to Ru–Ru scattering paths arising from [RuO_6] octahedra sharing an edge or a vertex, respectively. Despite the differing multiplicities of these paths (2 vs. 8, based on the crystallographic structure of RuO_2) their intensities appear comparable. Similar spectra were reported for partially hydrated RuO_2*nH_2O with n~0.29. The number of Ru neighbours estimated from the fit (Table S2) is significantly lower than that expected for bulk RuO_2, which is due to the presence of a fraction of very small RuO_2 particles. Notably, the Debye-Waller factors associated with the Ru-Ru scattering paths (0.004 Å^{-2}) indicate substantial structural disorder, supporting the hypothesis of nanoscale crystallites rather than amorphous phases.

.

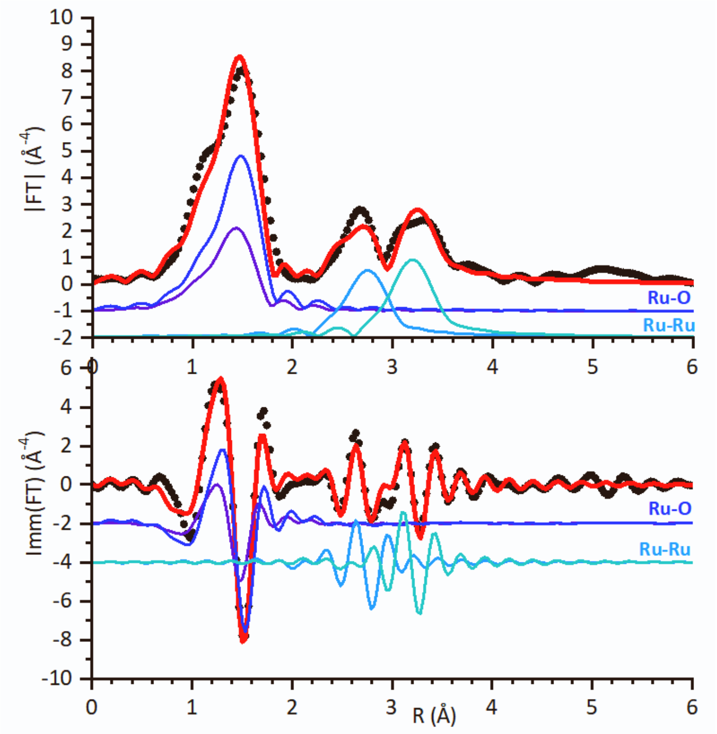

Figure S1. Phase-uncorrected |FT| and Imm(FT) of the k^3-weighted EXAFS spectrum of pristine Ru/Al2O3 (black dots), compared to the best fits (red). The two Ru-O and two Ru-Ru single scattering paths used contributing to the fit are also reported (vertically translated).

Table S2. Results of the fit of the Fourier-Transformed EXAFS spectrum of pristine Ru/Al2O3. R-factor = 0.045

Scatterer	DE (eV)	N_c (atoms)	R (Å)	σ^2 (Å^{-2})
O_1	-3 ± 2	1.8 ± 0.3	1.94 ± 0.01	0.006 ± 0.001
O_2		3.6 ± 0.6	1.99 ± 0.01	0.006 ± 0.001
Ru_1		1.1 ± 0.6	3.09 ± 0.02	0.005 ± 0.002
Ru_2		1.9 ± 1.0	3.56 ± 0.01	0.005 ± 0.002

2.3 Catalytic tests: solvent effect

Table S3. Solvent effect on the reductive upcycling of PE4000 promoted by the Ru/Al_2O_3 catalyst, in terms of both conversion and products selectivity. Reaction conditions: catalyst (Ru/Al_2O_3), solvent (H-donor solvents and THF), plastic (PE4000), temperature (220 °C), reaction time (60 min), initial Argon pressure (30 bar), stirring rate (500 rpm), vessel capacity (100 mL).

Solvent	Conversion ±Sd (%)	Products Selectivity (%)			Gases (%)	Wax[a](%)
		Gasoline (C5-C12)	Jet fuel & Diesel (C12-C20)	Lubricants (>C20)		
**2-PrOH**	65 ± 2	12	57	28	3	4
**EtOH**	42 ± 5	8	60	30	2	2
**MeOH**	37 ± 3	4	60	34	2	3
**THF**	3 ± 2	3	7	89	1	ND

[a]calculated from the sedimentation residue

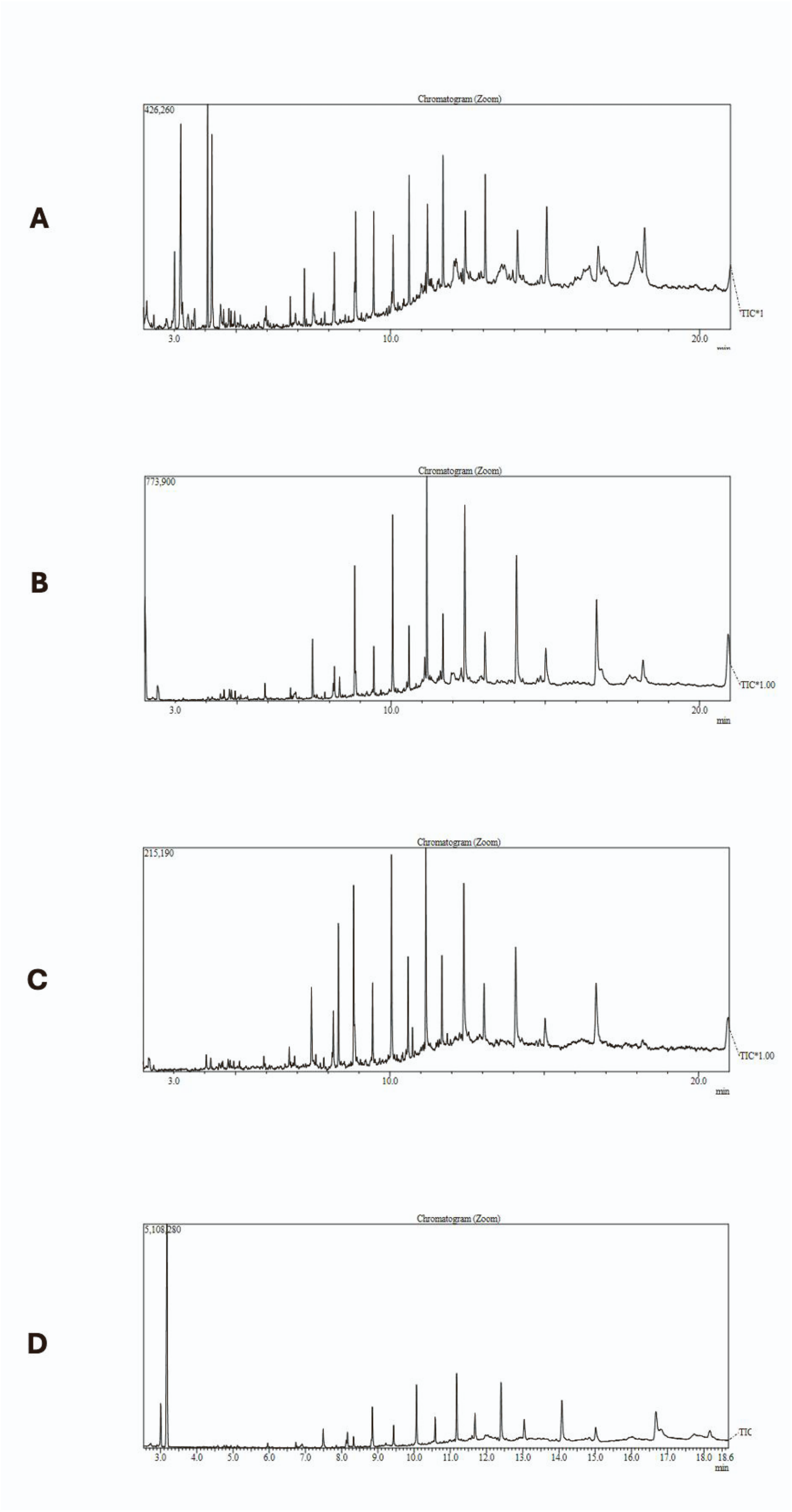

Figure S2. GC-MS signals of products obtained during the reductive upcycling of PE4000 with the Ru/Al_2O_3 catalyst, using different solvent A) 2-PrOH, B) EtOH, C) MeOH and D) THF. Reaction conditions: solvent, catalyst (Ru/Al_2O_3), plastic (PE4000), temperature (220 °C), reaction time (60 min), initial Argon pressure (30 bar), stirring rate (500 rpm), vessel capacity (100 mL).

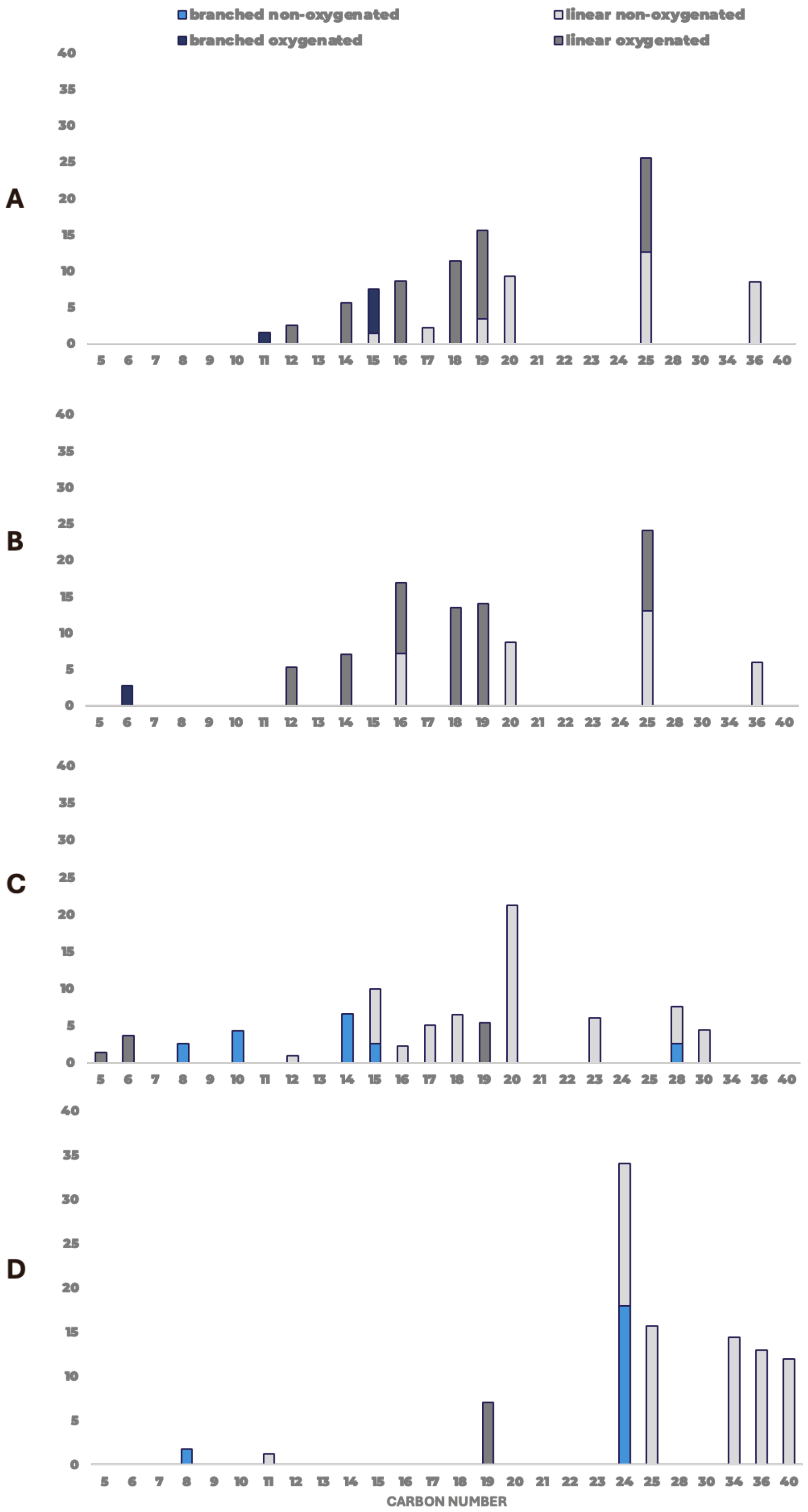

Figure S3. Product distributions using different solvent a) MeOH, b) EtOH, c) 2-PrOH, d) THF. The y-axis represents the percentage distribution of the detected products, calculated as the relative contribution of each identified product (grouped by carbon number) with respect to the total detected products. Reaction conditions: solvent, catalyst (Ru/Al_2O_3), plastic (PE4000), temperature (220 °C), reaction time (60 min), initial Argon pressure (30 bar), stirring rate (500 rpm), vessel capacity (100 mL).

2.4 Catalytic tests: effect of the gaseous atmosphere (H_2 vs Ar)

Table S4. Effect of 2-propanol and gaseous environment (H_2 vs. Ar) on the reductive upcycling of PE4000 promoted by the Ru/Al_2O_3 catalyst, in terms of both conversion and products selectivity. Reaction conditions: catalyst (Ru/Al_2O_3), solvent (2-PrOH), temperature (220 °C), reaction time (60 min), initial pressure (30 bar), stirring rate (500 rpm), vessel capacity (100 mL).

Conditions	Conversion ±Sd (%)	Products Selectivity (%)			Gases (%)	Wax[a](%)
		Gasoline (C5-C12)	Jet fuel & Diesel (C12-C20)	Lubricants (>C20)		
H_2 (no solvent)	20 ± 1	17	47	32	4	ND
H_2 (2-PrOH)	62 ± 4	17	56	25	3	ND
Ar (no solvent)	14 ± 3	9.7	66.6	19.7	3	ND

[a]calculated from the sedimentation residue

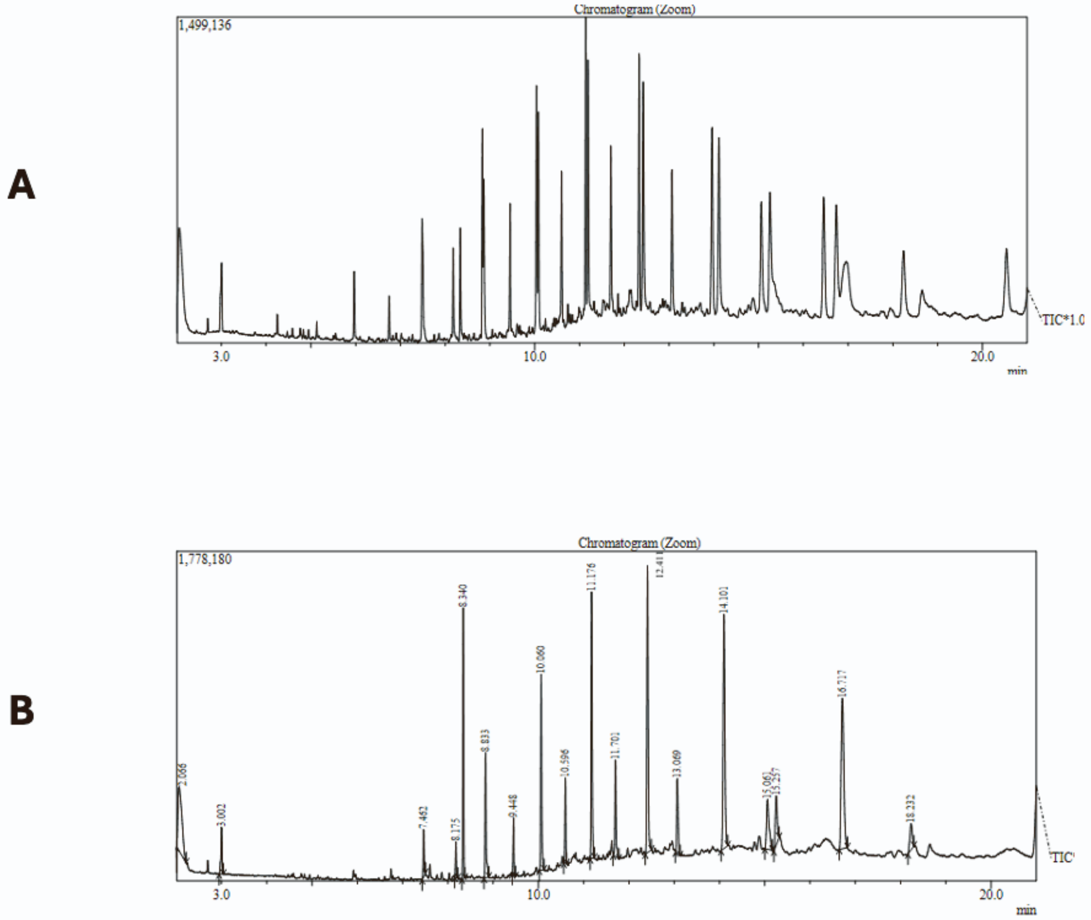

Figure S4. GC-MS signals of solid-solid state reactions under **A)** Hydrogen pressure (30 bar) **B)** Argon pressure (30 bar). Reaction conditions: catalyst (Ru/Al_2O_3), plastic (PE4000), no solvent, solvent used for liquid recovery (2-PrOH), Temperature (220 °C), reaction time (60 min), stirring rate (500 rpm), vessel capacity (100 mL).

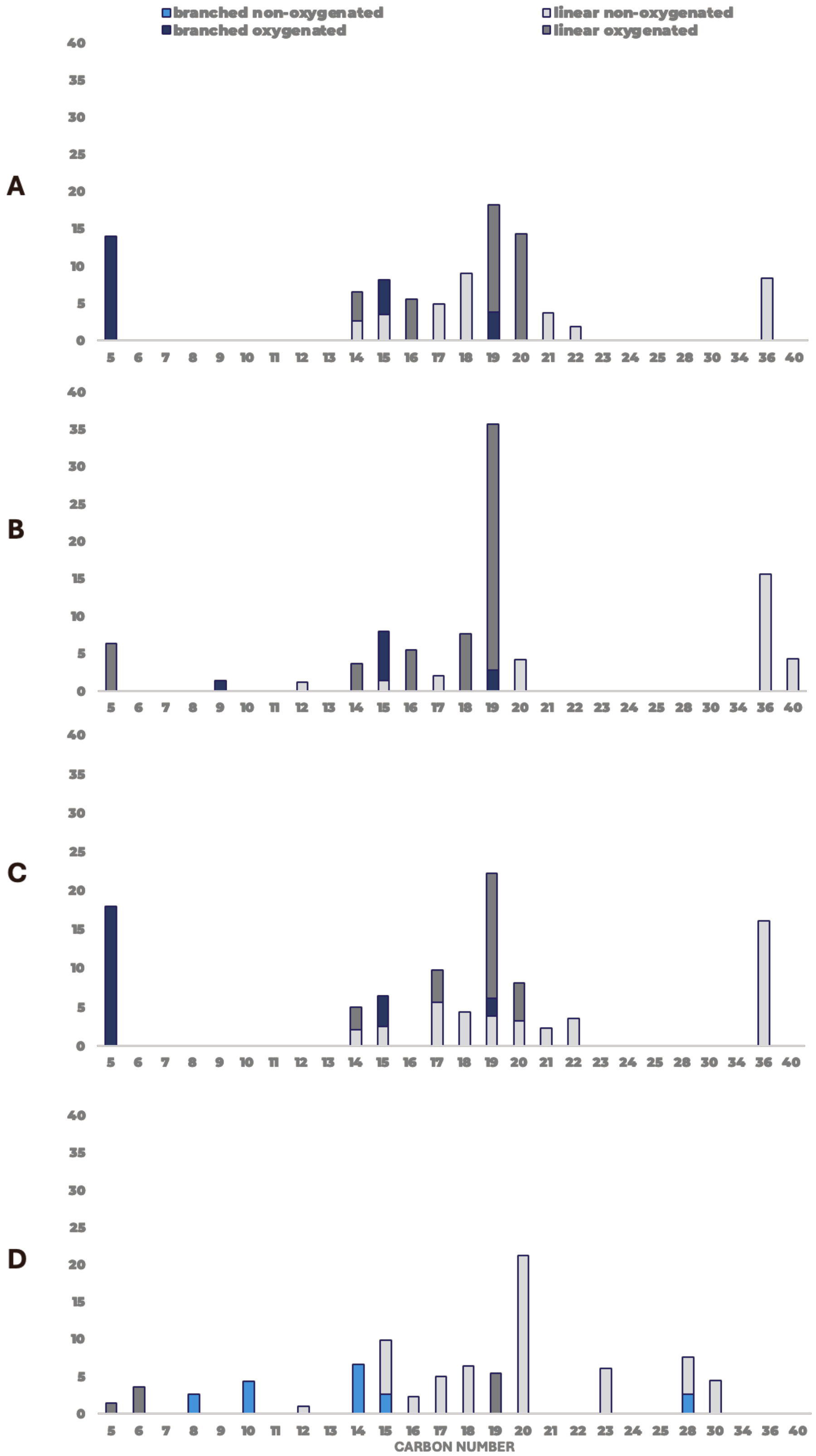

Figure S5. Product distributions of solid-solid state reactions under **a)** Hydrogen pressure (30 bar) **b)** Argon pressure (30 bar) and of solid-liquid state reaction using 2-PrOH under c) 30 bar of Hydrogen, d) 30 bar of Argon. The y-axis represents the percentage distribution of the detected products, calculated as the relative contribution of each identified product (grouped by carbon number) with respect to the total detected products. Reaction conditions: catalyst (Ru/Al_2O_3), plastic (PE4000), Temperature (220 °C), reaction time (60 min), stirring rate (500 rpm), vessel capacity (100 mL).

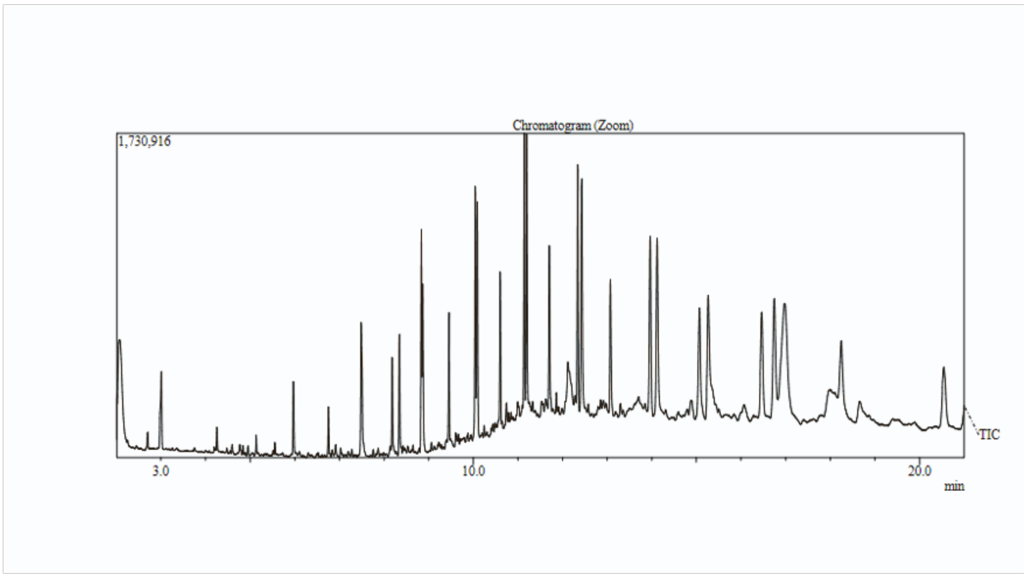

Figure S6. GC-MS signals of solid-liquid state reaction under 30 bar of hydrogen. Reaction conditions: catalyst (Ru/Al_2O_3), plastic (PE4000), solvent 2-PrOH, Temperature (220 °C), reaction time (60 min), stirring rate (500 rpm), vessel capacity (100 mL).

2.5 Catalytic tests: time effect

Table S5. Time effect on the reductive upcycling of PE4000 promoted by the Ru/Al_2O_3 catalyst, in terms of both conversion and products selectivity. Reaction conditions: catalyst (Ru/Al_2O_3), solvent (2-PrOH), temperature (220 °C), reaction time (3-12 h), initial Argon pressure (30 bar), stirring rate (500 rpm), vessel capacity (100 mL).

Time (h)	Conversion ±Sd (%)	Products Selectivity (%)			Gases (%)	Wax[a(%)]
		Gasoline (C5-C12)	Jet fuel & Diesel (C12-C20)	Lubricants (>C20)		
**3**	66 ± 5	15	57	24	4	ND
**6**	70 ± 1	16	58	21	5	ND
**12**	75 ± 2.55	19	63	14	5	ND

[a]calculated from the sedimentation residue

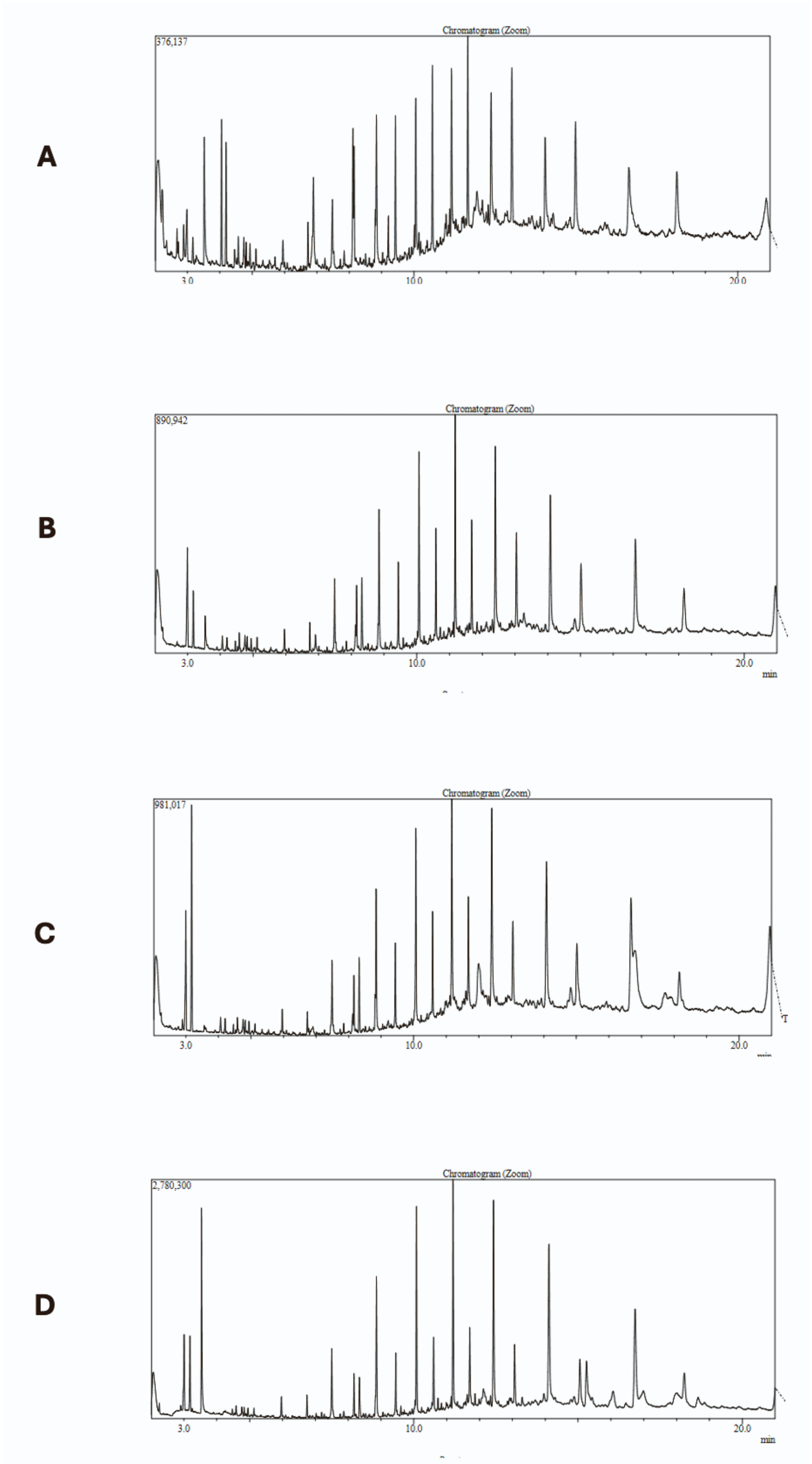

Figure S7. GC-MS signals of products using different reaction Time: A) 1 h, B) 3 h, C) 6 h, and D) 12 h Reaction conditions: catalyst (Ru/Al_2O_3), plastic (PE4000), solvent (2-PrOH), T (220 °C), initial Argon pressure (30 bar), stirring rate (500 rpm), vessel capacity (100 mL).

2.5 Catalytic tests: temperature effect

Table S6. Temperature effect on the reductive upcycling of PE4000 promoted by the Ru/Al_2O_3 catalyst, in terms of both conversion and products selectivity. Reaction conditions: catalyst (Ru/Al_2O_3), solvent (2-PrOH), temperature (200-240 °C), reaction time (60 min), initial Argon pressure (30 bar), stirring rate (500 rpm), vessel capacity (100 mL).

**Temperature (°C)**	**Conversion ±Sd (%)**	**Products Selectivity (%)**			**Gases (%)**	**Wax[a](%)**
		**Gasoline (C5-C12)**	**Jet fuel & Diesel (C12-C20)**	**Lubricants (>C20)**		
**200**	31 ± 7	9	48	42	1	5
**220**	65 ± 2	12	57	28	3	4
**240**	74 ± 4	22	57	17	4	<1

[a]calculated from the sedimentation residue

A

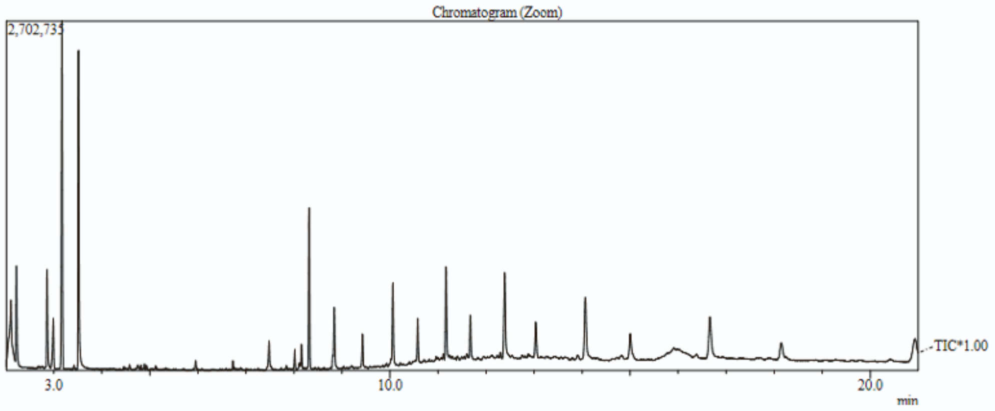

B

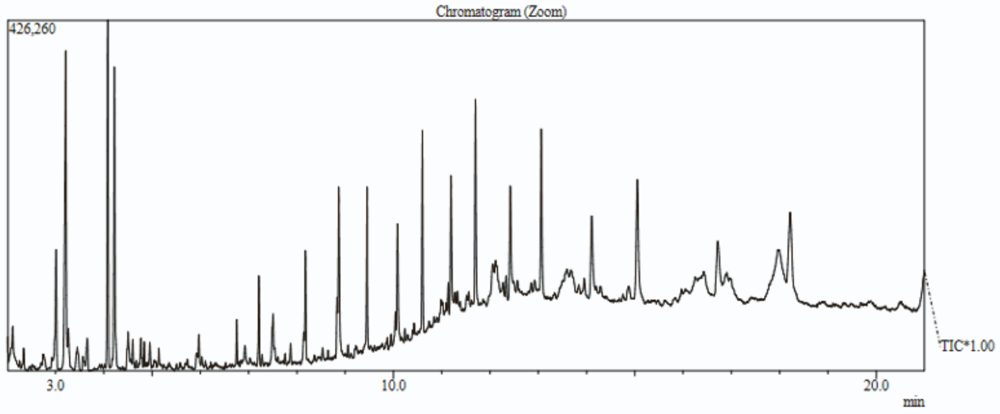

C

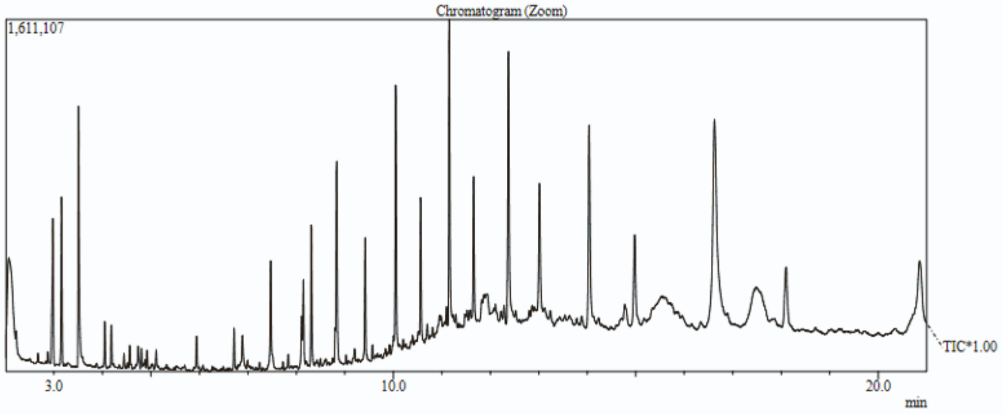

Figure S8. GC-MS signals of products using different reaction Temperature: A) 200, B) 220, and C) 240 °C. Reaction conditions: catalyst (Ru/Al_2O_3), plastic (PE4000), solvent (2-PrOH), reaction time (60 min), initial Argon pressure (30 bar), stirring rate (500 rpm), vessel capacity (100 mL).

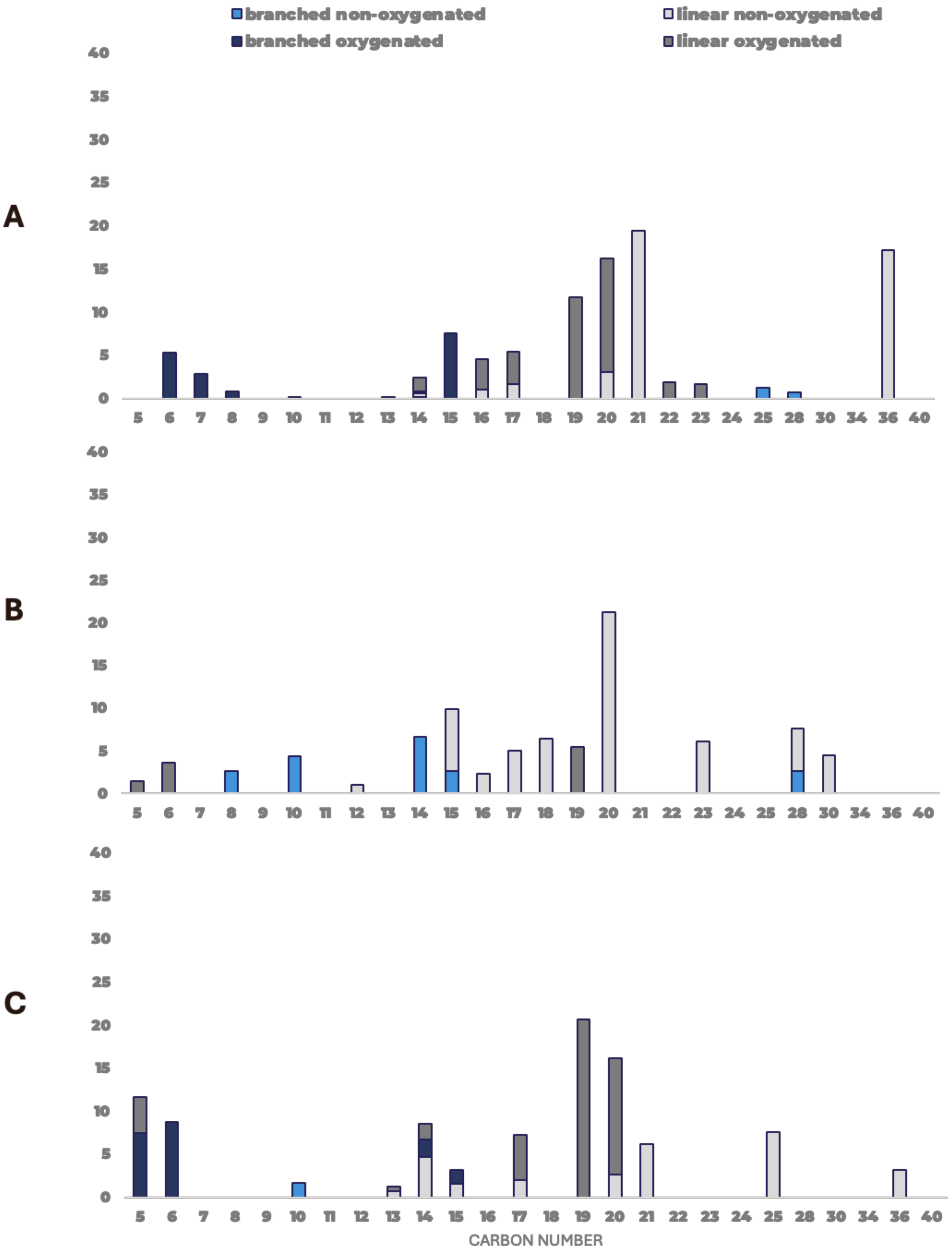

Figure S9.1 Product distributions using different reaction Temperature: a) 200, b) 220, and c) 240 °C. The y-axis represents the percentage distribution of the detected products, calculated as the relative contribution of each identified product (grouped by carbon number) with respect to the total detected products. Reaction conditions: catalyst (Ru/Al_2O_3), plastic (PE4000), solvent (2-PrOH), reaction time (60 min), initial Argon pressure (30 bar), stirring rate (500 rpm), vessel capacity (100 mL).

2.6 Catalytic tests: role of the substrate

Table S7. Conversion and products selectivity of the reductive upcycling of different POs promoted by the Ru/Al_2O_3 catalyst. Reaction conditions: catalyst (Ru/Al_2O_3), plastic, solvent (2-PrOH), temperature (220 °C), reaction time (60 min), initial Argon pressure (30 bar), stirring rate (500 rpm), vessel capacity (100 mL).

Temperature (°C)	Conversion ±Sd (%)	Products Selectivity (%)			Gases (%)	Wax[a(%)]
		**Gasoline (C5-C12)**	**Jet fuel & Diesel (C12-C20)**	**Lubricants (>C20)**		
Fresh Vegetable PE Bags	36 ± 6	17	31	48	4	<1
Tissue Wrapper	6.0 ± 1	24	31	41	4	ND

[a]calculated from the sedimentation residue

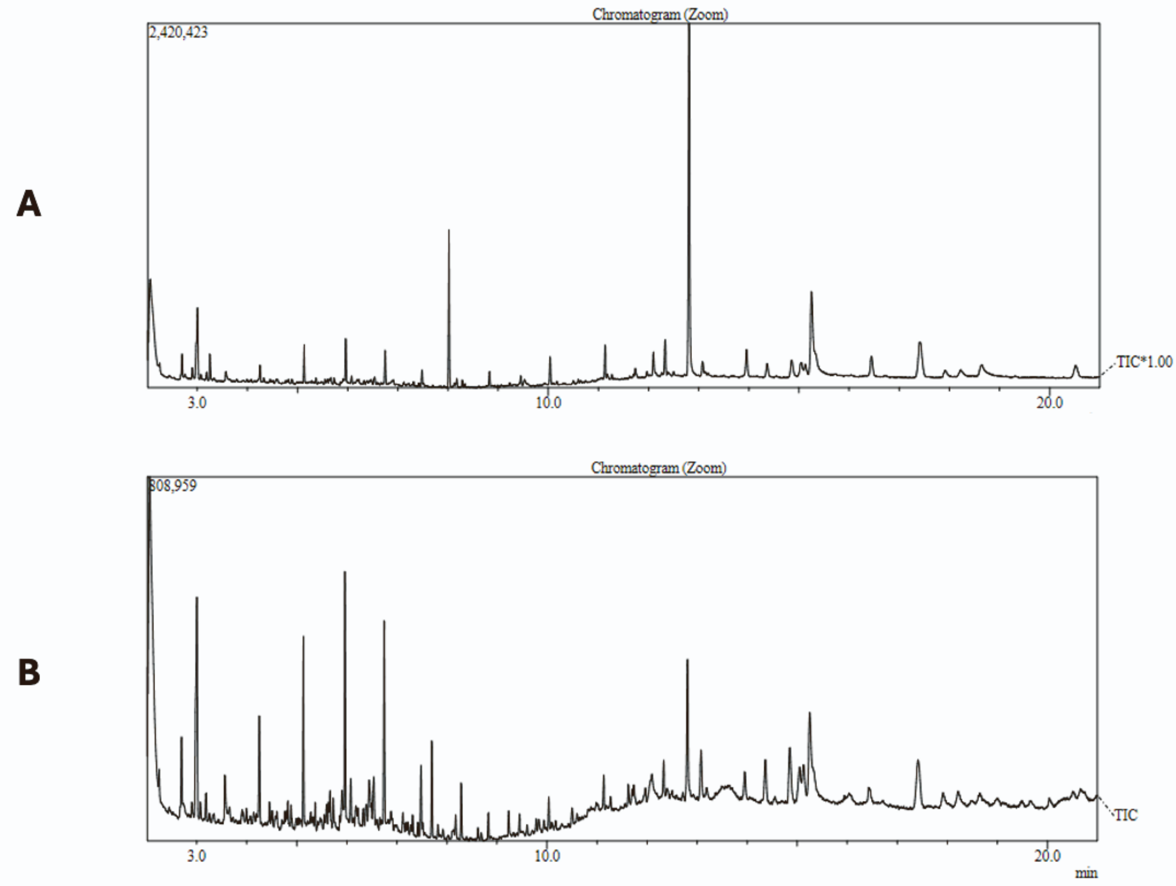

Figure S10 GC-MS signals of products using different substrates: A) Fresh Vegetable PE Bags, and B) Tissue Wrapper. Reaction conditions: catalyst (Ru/Al_2O_3), plastic, solvent (2-PrOH), temperature (220 °C), reaction time (60 min), initial Argon pressure (30 bar), stirring rate (500 rpm), vessel capacity (100 mL).

3 Supplemental References

1 M. Tamura, S. Miyaoka, Y. Nakaji, M. Tanji, S. Kumagai, Y. Nakagawa, T. Yoshioka, K. Tomishige, *Appl. Catal. B* **2022**, *318*, 121870.

2 J. E. Rorrer, G. T. Beckham, Y. Román-Leshkov, "Conversion of Polyolefin Waste to Liquid Alkanes with Ru-Based Catalysts under Mild Conditions "", JACS Au **2021**, *1*, 1, 8–12.

3 S. D. Jaydev, M.-E. Usteri, A. J. Martín, J. Pérez-Ramírez, "Identifying selective catalysts in polypropylene hydrogenolysis by decoupling scission pathways", Chem Catalysis **2023**, *3*, 100564.

4 T. Kwon, B. Ahn, K. H. Kang, W. Won, I. Ro, "Unraveling the role of water in mechanism changes for economically viable catalytic plastic upcycling", Nature Communications **2024**, *15*, 10239.

5 Y. Mei, J. Zhang, Z. Qu, F. Zeng, H. Jiang, Z. Tang, W. Xing, R. Chen, "Efficient Hydrogenolysis of Consumer-Grade High-Density Polyethylene Wastes over MgAl-Layered Double Oxide-Supported Ru Catalysts", *ACS Sustainable Chem. Eng*. **2024**, *12*, 49, 17914–17924.
